# Enhanced Electrochemiluminescence at the Gas/Liquid Interface of Bubbles Propelled into Solution

**DOI:** 10.1021/jacs.4c07566

**Published:** 2024-08-02

**Authors:** Sara Knežević, Joseba Totoricaguena-Gorriño, Rajendra Kumar Reddy Gajjala, Bruno Hermenegildo, Leire Ruiz-Rubio, José Luis Vilas-Vilela, Senentxu Lanceros-Méndez, Neso Sojic, Francisco Javier Del Campo

**Affiliations:** † University of Bordeaux, Bordeaux INP, ISM, UMR CNRS 5255, Pessac 33607, France; ‡ BCMaterials, Basque Center for Materials, Applications and Nanostructures, UPV/EHU Science Park, Leioa, Vizcaya 48940, Spain; § Grupo de Química Macromolecular, Universidad del País Vasco, UPV-EHU, Campus de Leioa, Vizcaya 48940, Spain; ∥ IKERBASQUE, Basque Foundation for Science, Bilbao 48009, Spain

## Abstract

Electrochemiluminescence (ECL) is typically confined to a micrometric region from the electrode surface. This study demonstrates that ECL emission can extend up to several millimeters away from the electrode employing electrogenerated chlorine bubbles. The mechanism behind this bubble-enhanced ECL was investigated using an Au microelectrode in chloride-containing and chloride-free electrolyte solutions. We discovered that ECL emission at the gas/solution interface is driven by two parallel effects. First, the bubble corona effect facilitates the generation of hydroxyl radicals capable of oxidizing luminol while the bubble is attached to the surface. Second, hypochlorite generated from chlorine sustains luminol emission for over 200 s and extends the emission range up to 5 mm into the solution, following bubble detachment. The new approach can increase the emission intensity of luminol-based assays 5-fold compared to the conventional method. This is demonstrated through a glucose bioassay, using a midrange mobile phone camera for detection. These findings significantly expand the potential applications of ECL by extending its effective range in time and space.

## Graphical abstract

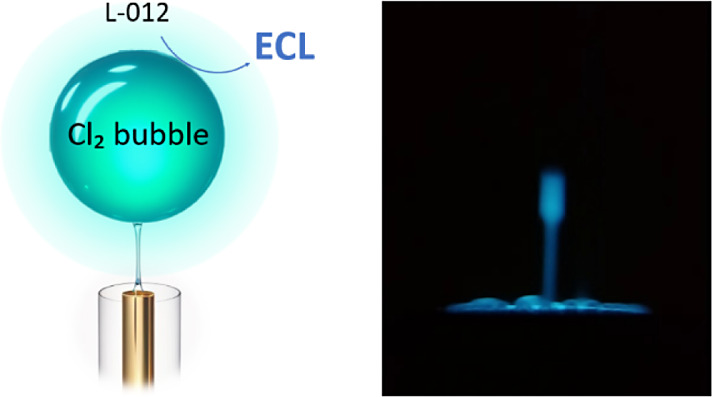

## Introduction

The gas/liquid interface is of fundamental interest in research and in the chemical industry. For instance, the gas/water interface of microdroplets shows unique redox chemistry with accelerated reaction kinetics.^1–6^ Bubble formation is highly relevant in the industrial production of gases such as hydrogen and oxygen by electrochemical water splitting, and chlorine in the chlor-alkali process.^7^ In the latter, chloride ions are oxidized at an anode, forming gaseous chlorine, one of the most important products of the chemical industry. However, bubbles have a notoriously bad press in electrochemistry because the generation of adherent bubbles at the electrode surface affects the efficiency of most electrochemical reactions. Indeed, bubbles block the electrode surface, preventing electron-transfer reactions and leading to drops in faradaic current and process efficiency losses. Therefore, gas bubbles are generally considered as detrimental in electrochemistry, particularly in electroanalysis.

Among the electrochemical techniques, electrochemiluminescence (ECL) is a light emission phenomenon confined to the immediate vicinity of an electrode surface.^8^ ECL is a very sensitive analytical technique based on orthogonal principles with an electrochemical trigger and an optical readout. Electrogenerated radicals react homogeneously in solution through a redox reaction with a luminophore and populate its excited state. ECL is very efficient for operando studies of chemical reactivity near electrode surfaces,^9–18^ in confined spaces,^19–24^ at interfaces^6,25–28^ and catalytic sites.^29–34^ Thanks to its high sensitivity, excellent spatiotemporal controllability and near-zero background signal, it has become a powerful tool in bioanalysis, immunosensing^12,20,35–41^ and imaging applications.^31,42–51^

Unfortunately, ECL is limited by relatively low light intensities, rapid signal decay, and spatial confinement to the micrometric region near the electrode surface.^52,53^ The limited lifetime of the electrogenerated species limits the spatial extension of the ECL-emitting layer.^9,22,54,55^ In this context, the purpose of this article is to improve the ECL method by amplifying the ECL emission and extending its duration in space and time. Here, we focus on the coreactant system typically used for bioanalysis, employing a derivative of luminol, 8-amino-5-chloro-2,3-dihydro-7-phenyl-pyrido­[3,4-*d*]­pyridazine-1,4-dione (L-012)^56,57^ as a luminophore, and hydrogen peroxide as a coreactant. Luminol (and its derivatives) emits light upon oxidation in the presence of H_2_0_2_ in alkaline aqueous solutions. The luminescence intensity is linearly dependent on the H_2_O_2_ concentration.^58–63^ The mechanism of this ECL generation is complex, involving luminol oxidation to yield diazaquinone radicals (L•−).^15,64^ These radicals subsequently react with hydrogen peroxide anion (HO_2_^–^) or other reactive oxygen species (ROS), namely hydroxyl radicals (OH^•^) and superoxide radicals (O_2_^•–^), produced during H_2_O_2_ decomposition, water oxidation,^65,66^ or reduction of dissolved oxygen,^64,67^ ultimately populating the excited state and emitting blue light upon decay.^15,64,68^

Enhancing the ECL intensity, duration, and spatial extension in the luminol/H_2_O_2_ coreactant system depends significantly on understanding the underlying mechanisms for ECL generation.^15,53^ Common strategies for modulating the luminol ECL response involve designing more efficient ECL emitters such as substituted luminol derivates,^56,69–71^ employing materials^64–67,72^ and experimental conditions^15,73^ that facilitate the generation of radical intermediates and using reagents that lead to prolonged ECL generation.^71,74^ Another approach toward accelerating the rates of chemical reactions and improving their efficiency involves conducting the reactions within confined environments,^20,23,72,75^ such as micropores,^15,76,77^ droplets,^26,27,78^ or interface^79^ of bubbles,^6,28,80^ leading to enhanced ECL intensities. Moreover, within microscopic bubbles and droplets, which possess a high surface-to-volume ratio, interfacial effects can significantly stimulate chemical reactivity, as already mentioned.^6,28,81^ For instance, Ciampi and coworkers demonstrated that the elevated self-ionization constant of water at the gas–water interface leads to the accumulation of hydroxide ions, resulting in the electrically charged corona of the bubble-promoting oxidative processes (specifically ROS generation) at the electrode-gas–water interface.^6^

Here, we implemented a simple experimental approach involving the electrochemical generation of chlorine gas bubbles on the surface of an anode to enhance the ECL emission of L-012 (Figure 1). In chloride-containing solutions, these bubbles predominantly consist of chlorine, which undergoes a reaction with water, yielding hypochlorite (ClO^–^). Hypochlorite present at the interface between the aqueous phase and the electrochemically generated bubbles can homogeneously oxidize L-012.^59,82^ Simultaneously, the bubble corona effect^1,6^ elevates the oxidation reaction rates at the electrode/gas/electrolyte interface, ensuring the generation of an abundant supply of ROS. ROS then react with the oxidized L-012, producing its excited state and resulting in an enhanced and long-lasting ECL emission at the bubble’s surface. This emission, driven by the *in situ* (electro)­chemical generation of oxidants, can extend up to 5 mm from the electrode surface after bubble detachment. While ECL is typically confined to processes within the immediate vicinity (a few micrometers) of the electrode, our method extends its application to phenomena occurring far away (up to several millimeters) from the electrode surface. In prospect, this remotely generated and long-lasting ECL can be used to analyze dynamic processes in the bulk that are inaccessible to traditional ECL methods.

**Figure 1:**
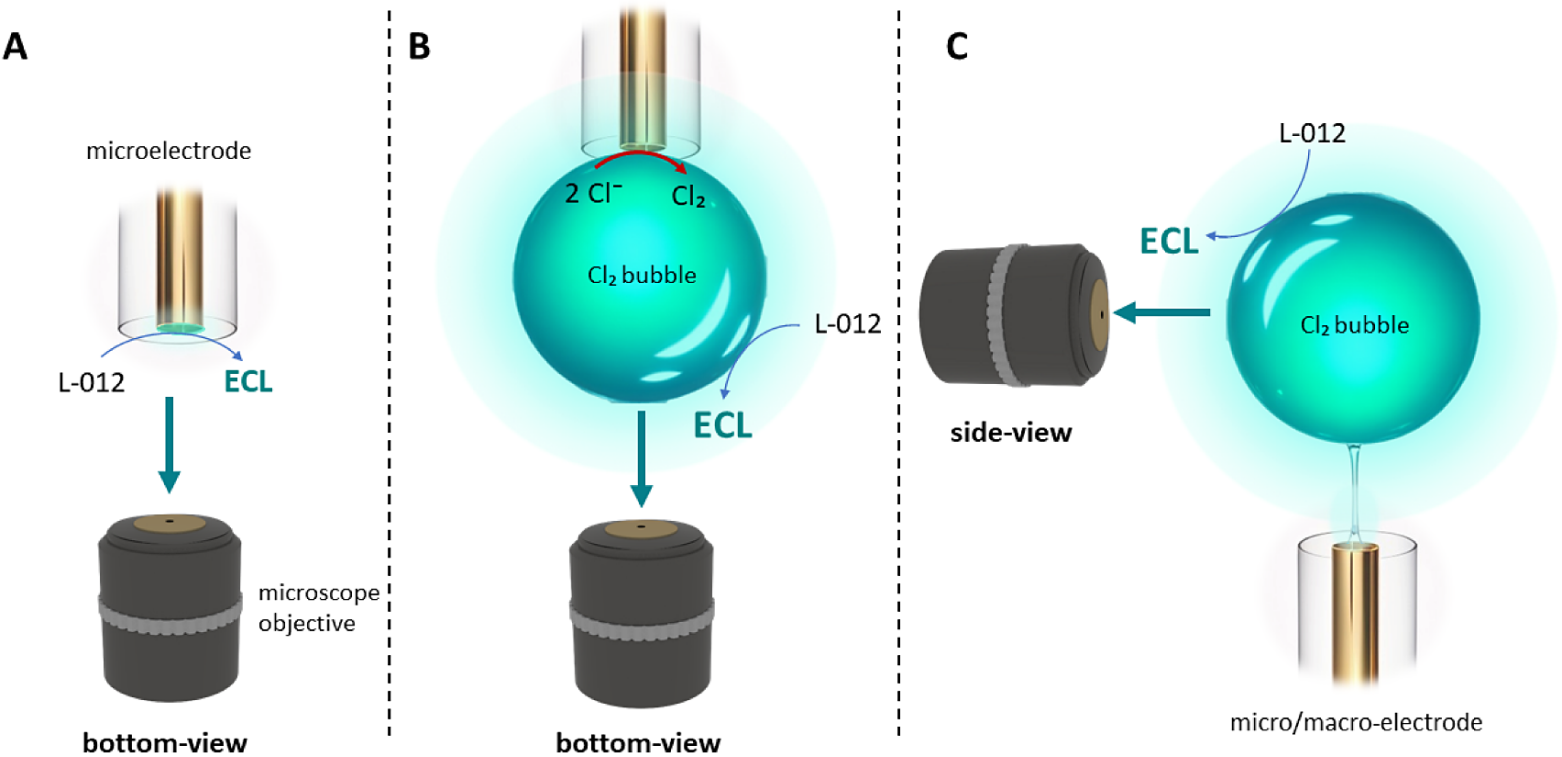
Schemes illustrating the L-012 ECL emission process: (A) at the surface of a gold microelectrode, (B) at the interface between the Cl_2_ bubble (gas phase) electrogenerated by a microelectrode and the aqueous phase before, and (C) after the Cl_2_ bubble detaches from the electrode surface. To study the ECL enhancement at the gas/liquid interface and the spatial extension (and propagation) of the ECL-emitting layer after bubble detachment, imaging experiments were performed in (B) bottom-view and (C) side-view configurations with the electrode positioned (A, B) above or (C) below the Cl_2_ bubble.

## Experimental Section

### Reagents and Apparatus

Reagents and solvents were purchased from commercial suppliers and used without further purification. L-012, hydrogen peroxide, glucose oxidase, α-d-glucose, benzoic acid, sodium dihydrogen phosphate dihydrate, disodium hydrogen phosphate, potassium chloride and potassium nitrate were purchased from Sigma-Aldrich and used as received.

All solutions were prepared with Milli-Q water of resistivity not less than 18 MΩ cm. A 25-μm gold disk microelectrode (ItalSens, PalmSens, NL) was used for single bubble imaging experiments, while 1.5 mm gold, 1.5 mm platinum and 3 mm glassy carbon disk electrodes from BASi (IN, US) were used for ECL imaging and experiments with a photomultiplier tube (PMT). All working electrodes were polished using 0.05 μm alumina polishing powder, followed by sonication in ethanol before each experiment.

### Electrochemistry and ECL

The experiments were performed in custom-made electrochemical cells with a glass slide window for collecting ECL signals. All the cells used a three-electrode configuration, in which a gold 25-μm disc microelectrode, a gold 1.5 mm disc electrode, a platinum 1.5 mm disc electrode or a glassy carbon (GC) 3 mm disc electrode acted as a working electrode, Ag/AgCl (LiCH_3_COO 0.1 M) or Ag/AgCl/KCl 3 M as reference electrodes, and a Pt wire as a counter-electrode.

For ECL and bright field microscopies, an epifluorescence microscope from Leica (DMI6000, Leica Microsystems) equipped with an ultrasensitive Electron-Multiplying Charge Coupled Device camera (EM-CCD C9100–23B from Hamamatsu, Hamamatsu Japan) was used with an inverted 10× microscope objective (10×/0.25, 17.6 mm; Leica, number 11506260). The integrated system also included a potentiostat (PalmSens 4) suitable to apply the required potentials to generate ECL. All micrographs were collected without using filter cubes.

An Autolab PGSTAT101 (Metrohm-DropSens) controlled by NOVA 2.1 was used for all other electrochemical measurements and the custom-made electrochemical cells were interfaced with either a photomultiplier tube (PMT, Hamamatsu R5070, with a Hamamatsu C9525 high-voltage power supply and a Keithley 6485 Picoammeter) or an Honor Magic 4 Lite 5G smartphone with an attached 12×/24× macro lens (Apexel, Amazon, ES). Videos were recorded in RAW format using MotionCam Pro for Android.

ECL measurements were conducted in 0.2 M phosphate buffer solution (PBS) at pH 8 with 0.05 M KCl or KNO_3_ supporting electrolyte, 0.5 M H_2_O_2_, and 0.42 mM L-012. The pH was adjusted using NaOH or H_3_PO_4_ to obtain the desired value. Cyclic voltammetry from 0 to 3 V with a scan rate of 0.1 V s^–1^ or chronoamperometry (CA) at 0.7 V, 2.4 or 3 V were used to generate the ECL emission. ECL was captured using an EMCCD with an integration time of 0.1 or 1 s.

## Results and Discussion

### Electrochemical and ECL Characterizations

The main aim of this study is to extend and improve the performance of ECL and to achieve more efficient emission without relying on nanomaterials or specific experimental setups to boost the ECL signal. To accomplish this objective, our approach relies on microelectrodes to electrogenerate Cl_2_ microbubbles, which surface triggers an intense ECL emission (Figure 1). First, we investigated the electrochemical reactivity of the L012/H_2_O_2_ system, especially at high anodic potentials, which are usually overlooked in electrochemical analysis. Figure S1A–C show the voltammetric responses in 50 mM KNO_3_ (red curves) or KCl (blue curves) supporting electrolyte in PBS (pH 8) at the surface of glassy carbon (GC), Au and Pt electrodes, respectively. At the GC electrode, electrolysis starts approximately at 0.1 V lower potential in KCl than in KNO_3_ supporting electrolyte (Figure S1A), indicating the involvement of two parallel processes in KCl: water oxidation and chloride oxidation,^83^ as opposed to a single process: water oxidation, that takes place in KNO_3_. Conversely, at the Au and Pt electrodes, anodic reactions are shifted to more positive potentials in the KCl-containing solution (Figure S1B,C). This is a consequence of the strong adsorption of chlorides at the electrode surface, which hindered OH^–^ adsorption and shifted gold and platinum oxidation to more positive potentials,^83,84^ while also promoting chlorine evolution at higher overpotentials. Thus, biasing Au, Pt and GC electrodes to high anodic potentials leads to oxygen evolution (OER) in the KNO_3_ electrolyte solution, whereas a dominant reaction in 50 mM KCl solution is chlorine evolution (CER).^83^ Under the same experimental conditions, we evaluated the influence of the supporting electrolyte on the oxidation of L-012 (insets in Figure S1A–C). L-012 oxidation resulted in a sharp peak with a half-wave potential of 0.65 V vs Ag/AgCl (all the potentials of this study are reported versus this reference electrode) in KNO_3_ and 0.85 V in KCl-containing solution, at both GC and Au working electrodes. The previously discussed chloride adsorption prevented the adsorption of other reactive species, in this case L-012, and caused this anodic shift, accompanied by a small decrease in the observed peak current. Conversely, L-012 oxidation does not seem to be significantly affected by the adsorption of chlorides on the Pt electrode. However, a comparison of current–potential plots in KNO_3_ (red lines) and KCl (blue lines) supporting electrolytes containing 0.42 mM (full lines) or no (dashed lines) L-012 (Figure S2A) shows that oxide layer formation at the platinum electrode shifts to higher potentials in the presence of chloride ions (from ca. 0.36 to ca. 0.61 V). Thus, L-012 oxidation effectively takes place at different electrode surfaces in KNO_3_ and in KCl.

Moreover, under the same experimental conditions, Figure S1D–F shows the evolution of ECL intensity with the working electrodes’ potential. They demonstrate that the low-potential ECL emission at GC and Au electrodes shifts anodically in the presence of chloride, which is consistent with the observed change in L-012 oxidation potential in different supporting electrolytes (inset in Figure S1A,B). Furthermore, the low-potential ECL intensity is enhanced 5.4-fold and 2.4-fold in the presence of chlorides at the GC and Au electrodes, respectively. This enhancement is likely caused by the electrochemically produced OER and CER intermediates facilitating the homogeneous oxidation of the ECL species, leading to enhanced emission. This is evidenced by an increase in the faradaic current at potentials above ca. 1 V (insets in Figure S1A–C), where, although kinetically sluggish, OER and CER reactions are thermodynamically feasible.^83,85^ Thus, the enhanced ECL emission in the Cl^–^-containing solutions indicates the involvement of chlorine species in the mediation of the ECL reactions. Additionally, at the Pt working electrode, a sharp, peak-shaped L-012 emission arises at ca. 0.55 V in the KCl solution (red line, Figure S1F). Conversely, in the absence of chloride ions, a low-intensity ECL emission persists over a broad potential range (ca. from 0.6 to 1.25 V). This result is consistent with an anodically shifted oxidation of Pt electrode surface in KCl (Figure S2A), supporting the crucial role of adsorption and oxidation of chloride ions on the (electro)­chemical and surface properties of Pt electrode and, consequently, ECL behavior of luminol-based ECL systems.

Similar conclusions can be drawn from the ECL emission at high anodic potentials (i.e., above 2 V), where CER and OER proceed at significant rates. Regardless of the working electrode’s material, high-potential ECL arises in the KCl containing solutions but is absent when KNO_3_ is used as the supporting electrolyte. This clearly indicates that inorganic chlorine species are involved in the underlying mechanism of ECL generation by homogeneously oxidizing L-012 and its intermediates. Differences in high-potential ECL intensities at GC, Au, and Pt electrodes can be attributed to the distinct kinetics of CER at different electrode surfaces.

Finally, we probed the ECL signal of the L-012/H_2_O_2_ system at GC, Au and Pt electrodes. We scanned the potential from 0 to 3 V in the 50 mM KNO_3_ (red curvess) or 50 mM KCl solution (blue curves) (Figure S1G–I, respectively). Notably, ECL emission intensities in the presence of H_2_O_2_ are increased by ca. an order of magnitude, relative to the same conditions in the absence of H_2_O_2_. At both GC and Au electrodes, the onset of low potential ECL shifted to ca. 200 mV higher values in the KCl solution compared to the KNO_3_ solution. This shift is attributed to differences in the oxidation potential of L-012 in different supporting electrolytes (insets in Figure S1A,B). Conversely, regardless of the electrolyte composition, only a faint low-potential ECL, shifted to higher overpotentials, was observed over a wide potential range at the Pt working electrode. This is likely due to the earlier onset of Pt oxidation in the presence of H_2_O_2_, which, as previously discussed, results in altered electrode surface properties and reduced reactivity toward L-012 oxidation.

At high electrode potentials, where Cl_2_ and O_2_ evolution reactions take place, strong ECL emission was detected at all three electrodes when using KCl as a supporting electrolyte. This high-potential ECL emission is comparable to the low-potential ECL observed at the GC electrode and is 2.2 and 3 times higher than the low-potential ECL emission at the gold electrode in the KNO_3_ and KCl supporting electrolytes, respectively. In contrast, when using KNO_3_ as a supporting electrolyte, no high-potential ECL was observed at the GC electrode, while a low level of light emission emerged from the Au and Pt electrodes. This difference is due to the electrogenerated Cl_2_ reacting with water to produce ClO^–^, a potent oxidant that efficiently mediates the homogeneous oxidation of L-012, resulting in strong ECL emission in solution. Conversely, while hydroxyl and superoxide radicals formed during water oxidation generate strong ECL upon reacting with L-012 radicals,^65^ they do not efficiently promote the oxidation of L-012. Moreover, the evolution of O_2_ competes with the electrochemical oxidation of L-012, quenching the high potential ECL by decreasing the concentration of electrogenerated L-012 radicals. Thus, ECL at high electrode potentials in the L-012/H_2_O_2_ system highlights the crucial role of electrogenerated Cl_2_ in mediating oxidation reactions that produce ECL radical intermediates.

Consequently, ECL can be generated by oxidizing L-012 at the electrode surface at moderate anodic potentials (ca. 0.7 V) and on the surface of the gas bubbles produced by chloride oxidation at high potentials (above 2.0 V). In the latter case, electrogenerated oxidants (Cl_2_ and ROS) mediate the ECL production at the surface of the bubbles by homogeneously oxidizing the ECL reagents.

### Imaging Spatial and Temporal Reactivity: ECL Emission Produced at the Electrode Surface versus at the Gas/Liquid Interface of Single Bubbles

Capturing the ECL signal with an optical microscope allowed us to spatially resolve the light distribution at different distances and in various phases from the electrode surface. The resulting optical information can then be correlated to the electrochemical responses. First, we recorded the ECL emission generated at the level of the surface of a microelectrode with a microscope in a bottom-view configuration (Figure 1A). Micrographs in Figure 2 show the ECL emission of L-012 in PBS solution (pH 8) containing KNO_3_ (Figure 2A–C) or KCl (Figure 2D–F) as supporting electrolytes. The gold microelectrode was biased (from left to right) at 0.7, 2.4, and 3 V. Figure 2A,D shows micrographs in KNO_3_ and KCl supporting electrolytes, respectively, when the potential of the working electrodes was set to 0.7 V. Both images were captured during the application of a constant potential with an exposure time of 1 s for the CCD camera. Applying such a moderate anodic potential resulted in the generation of stable ECL signals confined to the surface of the microelectrode. The size of the ECL-emitting layer corresponds to the dimension of the microelectrode. At this potential, no bubble was produced in either electrolyte. The mean ECL intensities were 2317 arbitrary scale units (a.u.) and 467 au in KNO_3_ and in KCl, respectively (see Figure 3A). The brighter ECL emission in the KNO_3_ electrolyte can be explained by the more efficient oxidation of L-012 at 0.7 V in the absence of chloride ions (see inset in Figure S1B).

**Figure 2:**
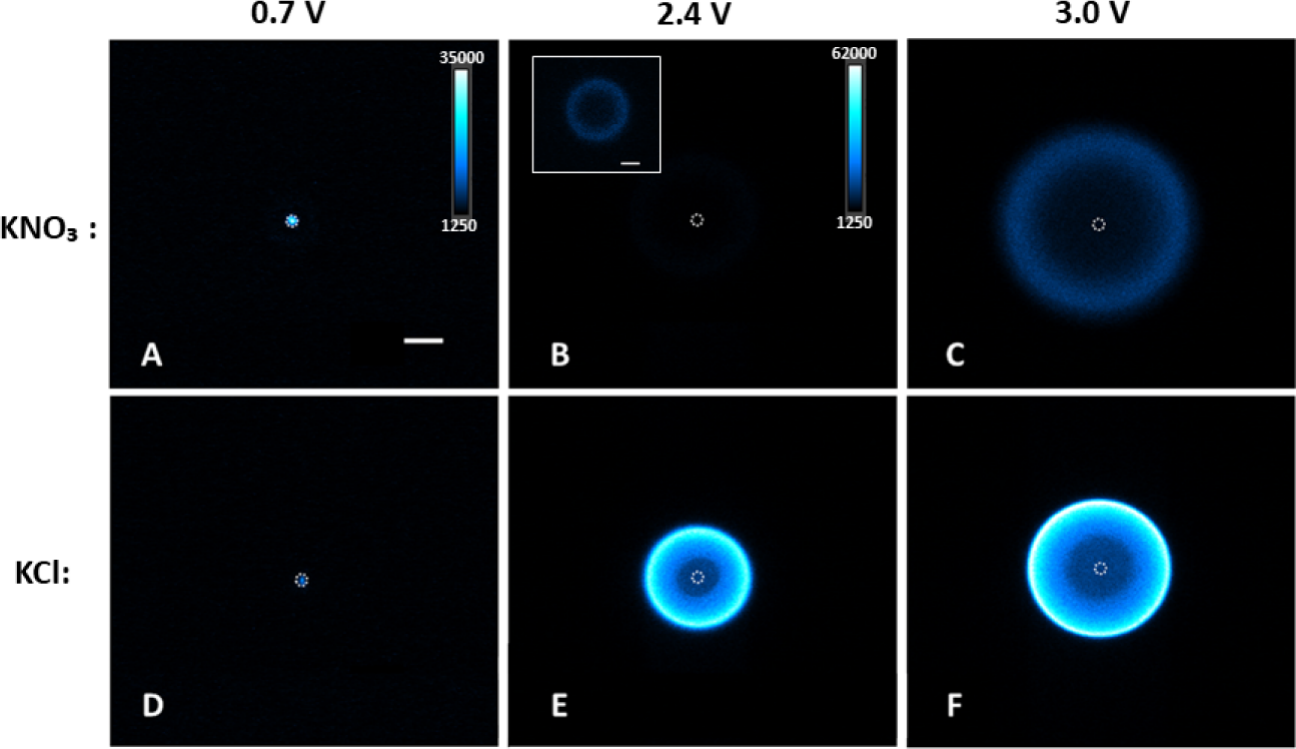
Bottom-view images of the ECL emission at the gold 25-μm disc microelectrode in the 0.2 M PBS solution (pH 8) containing 0.5 M H_2_O_2_, 0.42 mM L-012 and 50 mM (A–C) KNO_3_ or (D–F) KCl. Electrode potential was set to (A and D) 0.7 V, (B and E) 2.4 V and (C and F) 3 V vs Ag/AgCl for 20 s. Images A and D were recorded with a 1-s exposure time, upon applying the electrochemical potential for 1 s. Images (B, C, E and F) were taken with a 0.1-s exposure time, upon applying the potential for 5 s (when the Cl_2_/O_2_ bubbles were fully formed). False-color ECL images (A and D) and (B, C, E and F) were coded with the light intensity scales 1200–35 000 (shown in A) and 1200–62 000 (shown in B), respectively. The inset image in (B) was coded with the intensity range 1200–10 000. The dashed circle materializes the gold disc microelectrode. Scale bar: 50 μm.

**Figure 3:**
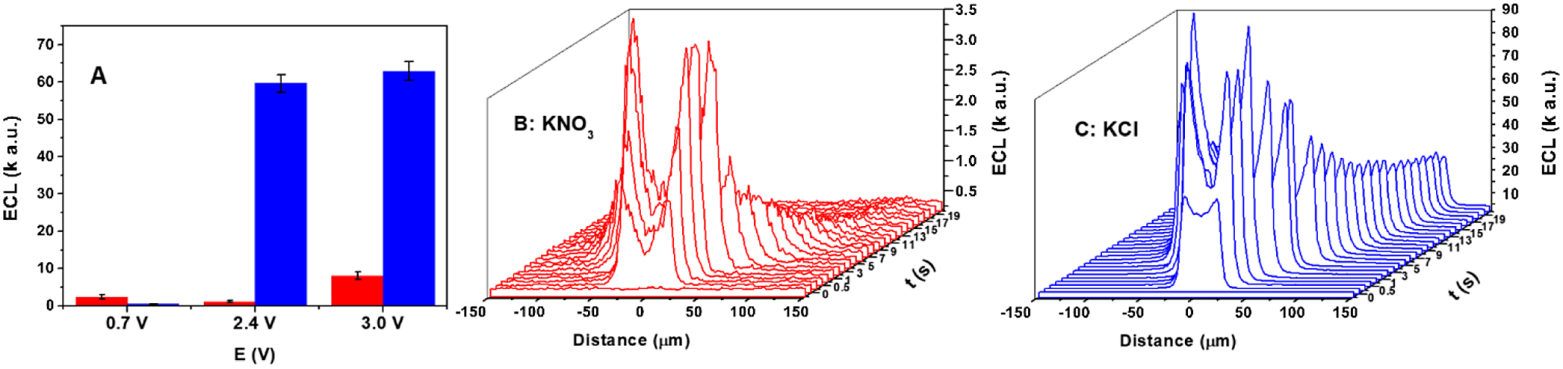
(A) The mean ECL intensity in the presence of 50 mM KNO_3_ (in red) or 50 mM KCl (in blue) supporting electrolyte upon applying chronoamperometric pulses of 0.7 V, 2.4 and 3 V vs Ag/AgCl. The values in the bar charts represent the mean of experiments per conditions as shown in Figure 1 micrographs performed in triplicate in the presence (in blue) and absence of Cl^–^ (in red).The error bars indicate ±1 standard deviation from the mean. (B, C) Evolution with time of the ECL intensity profiles at the interface of the O_2_ and Cl_2_ gas bubbles produced at the surface of a gold microelectrode. The electrode was immersed in the 0.2 M PBS solution (pH 8), containing 0.5 M H_2_O_2_, 0.42 mM L-012 and 50 mM of (B) KNO_3_ or (C) KCl and the potential of 2.4 V vs Ag/AgCl was applied for 20 s. (C) The mean ECL intensity in the presence of 50 mM KNO_3_ (in red) and 50 mM KCl (in blue) supporting electrolyte upon applying chronoamperometric pulses of 0.7, 2.4, and 3 V vs Ag/AgCl (the histograms correspond to the micrographs in Figure 1).

Biasing the microelectrode at higher anodic potentials resulted in the generation of gas bubbles at its surface. We imaged the ECL signal across the solid/liquid/gas interface of a single electrogenerated bubble, before and after its detachment from the microelectrode surface (Figure 1B,C, respectively). First, to analyze the bubble evolution on the electrode surface (Figure 1B), the working electrode faces downward and is imaged through a coverslip glass using the epifluorescence microscope. This configuration allowed us to analyze a single electrogenerated gas bubble, which remained stable at the electrode surface throughout the experiment, by looking from the bottom of the bubble during and after its formation, as electrostatic and buoyancy forces kept it attached to the microelectrode. Electrogenerated bubbles grow on the electrode surface until the rate of gas formation and that of its diffusion back into the solution equilibrate. After this, bubbles dissolve by diffusion which, in the case of pinned bubbles, is slowed down due to the presence of the electrode on one side.^86^

Water oxidation in absence of chloride ions generates an O_2_ bubble whereas a Cl_2_ bubble is produced in the electrolyte containing KCl. Both bubbles were visualized by bright field microscopy (Figure S3B,D). ECL images were captured with an exposure time of 0.1 s, 5 s after imposing the potential in PBS solution (pH 8) containing H_2_O_2_, L-012 and either 50 mM KNO_3_ (Figure 2B,C) or 50 mM KCl (Figure 2E,F). All micrographs recorded at high anodic potentials (i.e., Figures 2B,C,E, and F) are displayed with intensity scales adjusted to 1200–62000 color levels, except for the **inset** image in Figure 2B, which has the intensity range set to lower values of 1200–10000. This adjustment was necessary to visualize the ECL emission in the inset of Figure 2B. Figure 2B,C depict the ECL generated at the gas/liquid interface of the oxygen bubbles produced by applying electrode potentials of 2.4 and 3 V, respectively. The substantial increase in the ECL intensity with the increase in electrode potential, from 1149 au at 2.4 V to 8083 au at 3 V (see Figures 2B,C and[Fig fig3]A) suggests the dependence of the produced ECL on the rate of the oxygen evolution reaction (OER). Comparing a micrograph captured in the ECL conditions (Figure S3A) with a corresponding bright field image of the same bubble (Figure S3B) clearly shows that ECL emission arises from the gas/liquid interface, suggesting the involvement of chemical reactions at the bubble surface. Indeed, the size of the bubble observed in bright field mode corresponds to the size of the ECL-emitting bubble, even if the ECL intensity is low and that the intensity scale has to be adjusted to be visualized. These ECL-producing reactions likely include homogeneous oxidation of L-012 mediated by hydroxyl radicals,^64^ followed by its subsequent reaction with the superoxide radicals to populate the excited state. Since both OH^•^ and O_2_^•–^ radicals are OER intermediates, their involvement in the ECL generation rationalizes the increasing emission intensity with the increase in electrode potential. Furthermore, the corona effect of the bubble promotes the generation of OH^•^ radicals at the electrode/bubble/electrolyte interface, possibly further enhancing ECL generation through the homogeneous oxidation of L-012.^6^

Next, we tested the influence of the presence of chloride and the potential generation of Cl_2_ bubbles on the ECL emission. We recorded both bright field and ECL images of the Cl_2_ bubbles electrogenerated at 2.4 and 3 V in the PBS solution, containing KCl supporting electrolyte, H_2_O_2_ and L-012 (Figure 2E,F, respectively). The mean ECL intensities of the obtained micrographs were 59 585 au at 2.4 V and 62 900 au at 3 V (Figure 3A). Thus, in the presence of electrogenerated chlorine, we observed 51.8-fold (at 2.4 V) and 7.8-fold (at 3 V) ECL enhancements relative to the signals produced in the same conditions in the solution containing KNO_3_ supporting electrolyte, and a 127.6-fold enhancement (at 2.4 V) compared to the low potential ECL (at 0.7 V) in the same electrolyte solution (Figure 3A). Figure S3C,D, showing ECL and bright field micrographs of the same electrogenerated bubble, demonstrates that the ECL emission is produced by oxidizing L-012 (and H_2_O_2_) at the bubble/electrolyte interface according to the well-established mechanism of ECL generation in the presence of dissolved chlorine species.^82^ Briefly, dissolved Cl_2_ reacts with hydroxide ions (or water) to generate hypochlorite, a potent oxidant that efficiently mediates the oxidation of L-012. Oxidized L-012 then reacts with hydrogen peroxide or ROS, triggering consecutive chemical transformations that populate the excited state of the luminophore.

Similar considerations can be applied to the ECL emission in the absence of H_2_O_2_ (see Figures S4 and S5). Upon biasing the microelectrode to the potential of 0.7 V, ECL emission confined to the surface of the electrode was observed in both KNO_3_ and KCl supporting electrolytes (see Figure S4A,D) with ECL intensities of 21 au and 48 au, respectively (histogram in Figure S5). Thus, although visible, this ECL response is two and one ranges of magnitude lower in KNO_3_ and KCl supporting electrolyte, respectively, when compared to the same conditions in the presence of H_2_O_2_. Furthermore, when biasing the microelectrode at high anodic potentials, the resulting ECL emission appeared at the outlines of the produced O_2_ and Cl_2_ gas bubbles (Figures S4B,C,E and F) with the intensities of 19 au and 42 au in KNO_3_ and 81 au and 64 au in KCl supporting electrolyte at 2.4 and 3 V, respectively (bar chart in Figure S5). The enhanced ECL at the interface can be attributed to the increased production of hydroxyl radicals at the gas/solid/liquid interface due to charge separation at the surface of the bubble. There are reports of this reaction occurring spontaneously at the gas/liquid interface of microdroplets, ultimately producing H_2_O_2_ trough recombination of hydroxyl radicals produced at the interface.^87,88^ Furthermore, as previously discussed, the gas/solid/liquid interface at the anodically polarized electrodes further enhances the production of ROS,^6^ demonstrating the importance of the interface effects on the reactivity within this heterogeneous system. The ECL intensity at the bubble’s interface was more significantly enhanced in the presence of chloride ions, further confirming the key role of electrogenerated chlorine for the L-012 ECL enhancement, while the enhanced ECL emission with the increase of electrode potential in KNO_3_ supporting electrolyte confirmed the importance of OER intermediates (i.e ROS) in producing the electrochemically excited state of L-012. Finally, the ECL intensity at high anodic potentials in the absence of H_2_O_2_ is lower by three and 4 orders of magnitude in KNO_3_ and KCl supporting electrolytes, respectively, compared to the same experimental conditions in the presence of 0.5 M H_2_O_2_. Thus, ECL in the absence of H_2_O_2_ would not interfere with any potential analytical application of such ECL measurements as it is in the acceptable error range and the ECL intensity of L-012 demonstrates high dependence on the concentration of H_2_O_2_.

Moreover, in the general case, ECL generation is confined to the immediate vicinity of the electrode surface, due to the limited lifetime of the electrogenerated species in solution, typically a few micrometers, depending on luminophore type, the experimental conditions and the operating ECL mechanisms.^9,11,12,15,22,40,54,89,90^ However, the here-reported approach demonstrates the possibility of extending the ECL-emitting region far from the electrode. Indeed, the sizes of the ECL-emitting bubbles shown are 138 and 186 μm (Figure 2E,F, respectively). This is an important result because it considerably extends the ECL-active region using electrochemical reactions that are generally considered detrimental.

Besides emission intensity, the generated signal stability and its duration are also important to most ECL applications. We analyzed the ECL intensity in KNO_3_ or KCl supporting electrolytes (in the presence of L-012 and H_2_O_2_) when the Au microelectrode was biased at 2.4 V for 20 s (Figure 3B,C, respectively). To ensure a good temporal resolution, we recorded a video during the 20 s potential pulse, at a rate of 8 frames per second. We then analyzed each frame by extracting intensity profiles of a rectangular region along the diameter of the electrogenerated bubbles (as shown in Figure S6A) and plotted the obtained profiles against time (Figures 3B,C). This allowed the analysis of ECL intensity during the bubble evolution at the electrode surface. Figure 3B shows that the ECL intensity initially increases with an increase in the diameter of the electrogenerated oxygen bubble, to reach a maximal value of ca. 3400 au, 5 s after initiating a potential pulse at 2.4 V, followed by a sharp decrease of the ECL emission with the increase in the bubble size, reaching the level of background noise at around 13 s after the potential pulse. Conversely, while ECL emission in KCl solution expresses a maximum of 90 000 au 5 s after imposing the potential, the decrease in the light intensity with the increase in the Cl_2_ bubble size is less pronounced, with the emission reaching a steady state at an emission intensity of ca. 40 000 au after 7 s, which lasted to the end of the 20 s chronoamperometric pulse (Figure 3C).

The increase in ECL intensity observed during the initial 5 s of the potential pulse, followed by an ECL decrease, is noteworthy as it indicates a common underlying phenomenon controlling ECL emission during bubble formation in both KCl and KNO_3_ electrolytes. Since the gas evolution reactions at 2.4 V are expected to selectively generate chlorine and oxygen in KCl and KNO_3_, respectively, this trend is not attributed to a shared reaction step in the two electrolyte solutions. Rather, it is linked to a general increase in electrochemical reactivity at the solid/gas/liquid interface, driven by the corona effect of the bubble.^6^ Initially, elevated reactivity at the interface compensates for the decrease in electrode surface area that is covered by the gas bubble. However, after the bubble reaches a certain size (at 5 s), the enhancement in electrochemical reactivity can no longer compensate for the large portion of the microelectrode isolated from the solution. Consequently, ECL generated at the surface of the O_2_ bubble rapidly decreases as ROS are no longer efficiently produced. In contrast, ECL at the Cl_2_ bubble reaches a plateau, as electrogenerated chlorine species can effectively mediate all the redox reactivity in the system, sustaining ECL emission even in the absence of ROS.

Several works have been devoted to the development of chemiluminescent and ECL systems that produce luminescence for a long-lasting time.^71,74^ Indeed, in the ECL field, since the species producing the excited state are electrogenerated, stopping their production ends the ECL emission instantly. Therefore, developing a long-lasting ECL emission can be considered as a challenging task. To investigate the temporal characteristics of the reported approach, we followed the ECL emission after stopping the imposition of the anodic potential (Figure S7). As expected, no ECL was observed in the KNO_3_ supporting electrolyte after stopping the application of the anodic potential (Figure S7A), and the system returns to its background value immediately. The behavior is completely different in the presence of KCl: ECL emission persists at the interface of the Cl_2_ bubble even when the potential is no longer applied to the microelectrode (Figure S7B–D). The intensity of this “afterglow” ECL exponentially decreases over time, vanishing after approximately 145 s (Figure S7E). This very long-lasting ECL is a very important result because the ECL process is based on a different mechanism involving the production of Cl_2_ bubbles. Moreover, the observed ECL emission layer expands into the solution, following the diffusion pattern of dissolved Cl_2_. We observed complex ECL patterns with bright ECL rings that are visible inside the Cl_2_ bubble in Figure S7B–D. Layman and Dick reported the reflection of ECL light (generated at the electrode surface) by CO_2_ bubbles located far away.^28^ The ECL patterns that we observed might be related to the reflection of the ECL emitted in solution by the glass insulating the Au microelectrode tip, and not from the ECL emission inside the bubble.

### ECL in Bulk: Mapping Spatial ECL Extension

As discussed above, bubble effects can significantly enhance the electrochemical reactivity on the solid/gas/liquid interface, amplifying drastically the resulting ECL intensity and its duration. In addition, once the Cl_2_ bubble is generated, ECL is emitted at the gas/liquid interface with the solution even in the absence of the applied potential. Consequently, after detaching from the electrode, the bubble is likely a reservoir for oxidizing species, initiating redox reactions and producing ECL emission while traveling through the solution. To confirm this, we followed the bubble formation and its subsequent detachment from the electrode in an upright experimental setup (Figure 1C). In other words, the working electrode faced upward in an electrochemical cell and ECL was imaged through a coverslip glass window on the side of the cell. In this arrangement, the buoyancy force propelled the bubble upward through the solution. To monitor the bubble evolution in solution and the volumic extension of the ECL-emitting zone at the bubble from the side, we changed the angle of observation with an orthogonal side-view configuration (Figure 1C). It supplements the bottom-view configuration with a 2D ECL imaging approach normal to the electrode surface and allowing to explore events occurring away from the electrode surface.

We used a 3 mm GC disc electrode, which ensured the bubbles would grow unrestrained by the limited electrode surface area of a microelectrode. This arrangement allowed us to observe the growth of the electrogenerated bubbles to their critical size and to track their path as the buoyancy force propelled them upward through the solution. We recorded a video (see Supporting Information) at 30 frames per second, while scanning the potential imposed on the working electrode from 0 to 3 V at 0.1 V s^–1^. The frames in Figure 4A–F show the vertical movement of a single Cl_2_ bubble from the electrode surface where it is electrogenerated into the bulk solution. These images reflect the change in size and velocity of the chlorine bubble. Furthermore, they show the diffusion pattern of dissolved chlorine (luminescent regions tracing the bubble’s path). Thus, the evolution of chlorine at high anodic potentials not only extends the ECL emission of L-012 far from the electrode surface, but it provides a means of visualizing the dissolution of gases, diffusion of chemical species, and reactions at phase interfaces and in solution.

**Figure 4:**
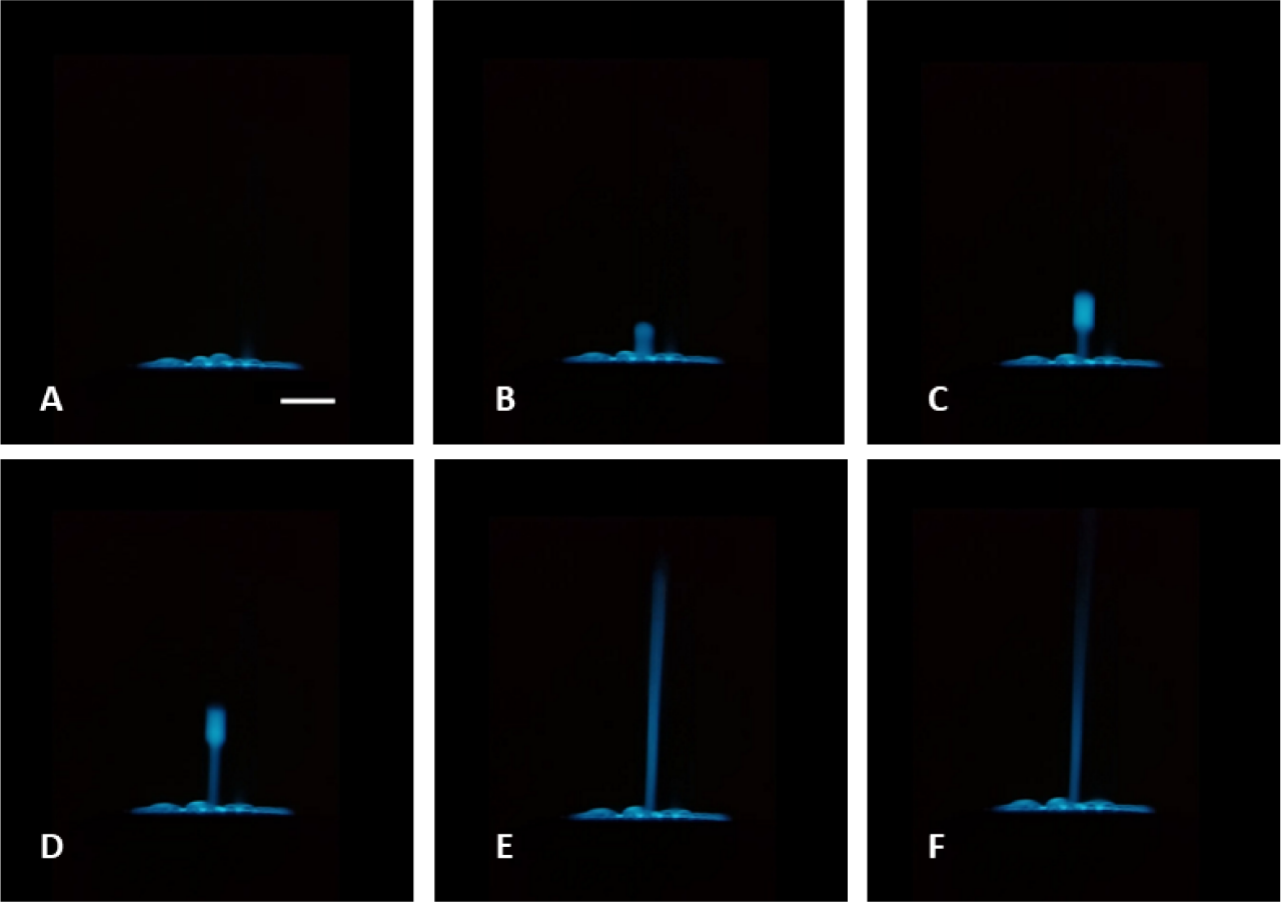
Side-view ECL images (A-F) depict the trajectory of a single electrochemically generated Cl_2_ bubble at different times (A–F: 0, 33, 67, 100, 233, and 367 ms, respectively) after detachment from the surface of the glassy carbon electrode. These images were extracted from a video provided in the SI, recorded during a cyclic voltammetry scan from 0 to 3 V at a scan rate of 0.1 V s^–1^ in a 0.2 M PBS solution (pH 8), containing 0.5 M H_2_O_2_, 0.42 mM L-012, and 50 mM KCl. Scale bar: 1 mm.

Furthermore, we evaluated how ECL intensity varied with distance from the electrode after the bubble shown in Figure 4 was propelled into the solution. This involved analyzing ECL intensity profiles along rectangular regions extending from the electrode surface into the bulk solution (see Figure S6B). Subsequently, we plotted these intensity profiles against time, as illustrated in Figure 5. The profiles from 0 s to 100 ms correspond to the images in Figure 4A–D. They show that the front of ECL emission follows the bubble propagation, with strong intensity concentrated on the bubble’s surface and comparable intensity values in the solution between the bubble and electrode. In other words, considering the electrogenerated bubble, the maximum ECL intensity is located in the regions far from the electrode, where the bubbles propel and meet “fresh” reactants. It indicates that ECL emission results from reactions occurring with chlorine without any additional involvement of the electrode reactions. However, after 100 s, the dissolution of chlorine caused the bubble to collapse, resulting in the strong ECL emission in solution and the disappearance of the sharp emission front. This ECL in solution reached a maximum distance of 5 mm from the electrode surface at 233 ms. At this point, the bubble was completely dissolved (Figure 4E). As the dissolved chlorine species were consumed, the ECL front gradually decreased until the ECL emission was no longer visible.

**Figure 5:**
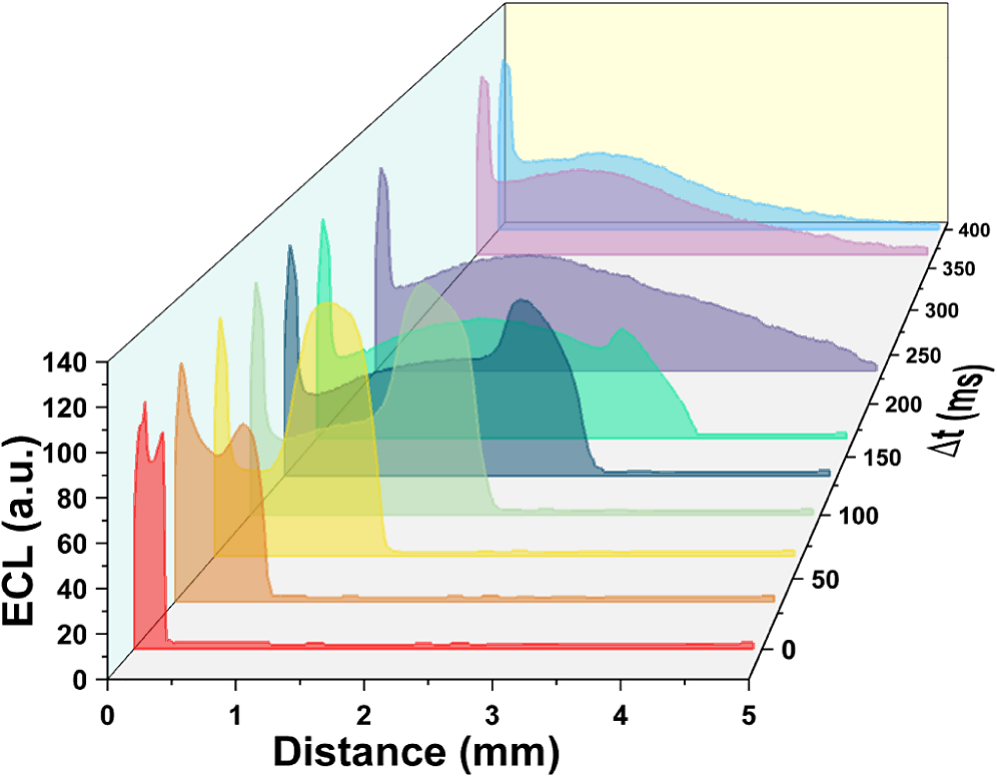
Spatial extension of the ECL emission following a detachment of a Cl_2_ bubble from the electrode surface (corresponding images are shown in Figure 3). The propagation of ECL up to 5 mm from the electrode indicates the possibility for delayed formation of the reactive radical species upon dissolution of the gas entrapped inside the bubble.

It is noteworthy that this is the first example of generating ECL emission a few millimeters from the electrode surface. As already mentioned, ECL phenomenon is usually confined to the micrometric region in the immediate vicinity of the electrode surface.^9,11,12,15,22,40,54,89,90^ The reported approach allows analyzing processes occurring far away from the electrode surface. Finally, while examples of ECL emission at the interface of two immiscible liquids exist in the literature,^25,26^ this study presents the first example of ECL produced at the gas/liquid interface.

### High Potential ECL Toward Enhanced Biosensing Performance

One of the key analytical applications of the luminol/H_2_O_2_ system is in biosensing,^20,35,38,91,92^ primarily due to the direct correlation of its ECL intensity with the concentration of hydrogen peroxide. Since H_2_O_2_ is commonly produced in enzymatic reactions involving oxidase enzymes, ECL emission by luminol can be used to effectively quantify substrates of such enzymes (e.g., cholesterol and glucose), or to study the kinetics of enzymatic reactions.^61,93^ Consequently, we anticipate that the enhanced ECL at the surface of the electrogenerated chlorine bubble, observed in here-investigated approach, would lead to a decrease in the detection limit in oxidase-based enzymatic sensors. Moreover, this robust ECL emission could enable the at-home monitoring of glucose using disposable ECL sensing devices, with detection facilitated by a smartphone camera.

In this proof-of-principle study, we used a phone camera to record the ECL emission in 0.2 M PBS solutions containing 0.45 mM LO12 and 1 mM glucose, immediately upon adding 1 mg mL^–1^ glucose oxidase. The images were captured when the working electrode was biased at 0.7 V (Figure 6A,C) and 2.4 V (Figures 6B,D) for 10 s, while 50 mM KNO_3_ (Figures 6A,B) or KCl (Figures 6C,D) served as the supporting electrolytes. Figure 6A,C shows that the ECL produced at low electrode potentials is not significantly influenced by the nature of the supporting electrolyte, and it exhibits average intensities of 2040 au and 1803 au (Figure 6E) in KNO_3_ and KCl supporting electrolytes, respectively. Conversely, upon applying a potential of 2.4 V to the working electrode, no ECL was detected in the KNO_3_-containing solution (Figure 6B), while bright ECL emission with an intensity of 9396 au emerged from the KCl-containing solution (Figure 6D and bar chart in Figure 6E). Thus, the ECL signal at 2.4 V was 5.2-fold higher than that observed at 0.7 V (Figure 6C), potentially facilitating more sensitive glucose detection and quantification. Moreover, Figure 6D reveals nonuniform ECL distribution at the electrode surface, with brighter regions corresponding to the localization of electrogenerated bubbles, further emphasizing the crucial contribution of chlorine bubble formation in the enhanced ECL for enzymatic detection of glucose.

**Figure 6:**
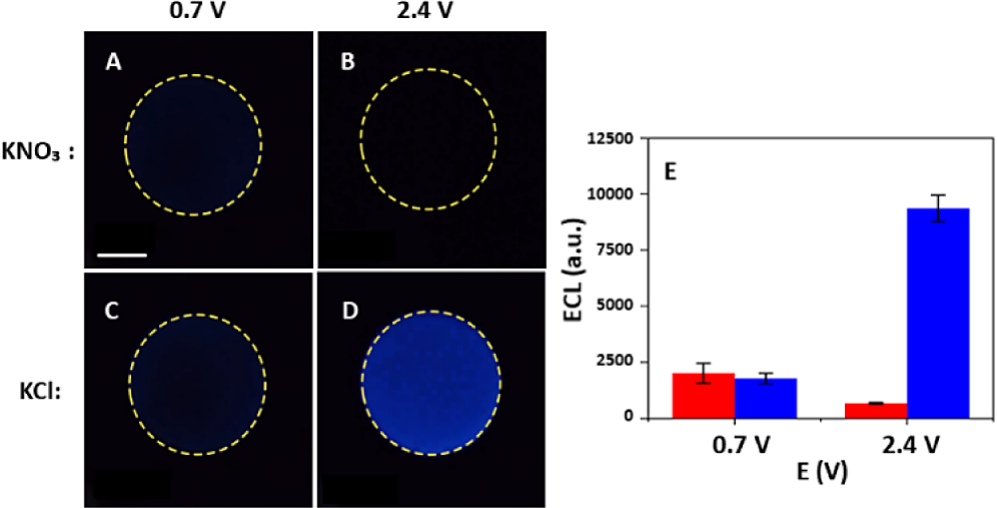
ECL detection of glucose. ECL emission on GC electrode in a 0.2 M PBS solution (pH 8) containing 0.42 mM L-012, 1 mg mL^–1^ glucose oxidase, and 1 mM glucose, with 50 mM (A, B) KNO_3_ or (C, D) KCl supporting electrolyte upon applying a 10 s potential pulse at (A and C) 0.7 V and (B and D) 2.4 V. Scale bar: 1 mm. (E) Bar chart showing the mean ECL Intensity extracted from the experiments performed in triplicate under experimental conditions as in (A–D) in the presence (in blue) and absence of Cl^–^ (in red) at different potentials. The error bars indicate ±1 standard deviation from the mean.

Control experiments performed under the same conditions, but in the absence of glucose show that ECL response is low and similar in all experimental conditions regardless of the value of applied potential or the composition of the supporting electrolyte (Figure S9). The intensity of such ECL signal in the KCl-containing solution at 2.4 V is 17.8 lower than in the same conditions in the presence of 1 mM glucose. This indicates that, while the effects of gas/liquid interface (including bubble corona effect) have a significant influence on (electro)­chemical reactivity and mechanism of ECL generation in the L-012/H_2_O_2_ system, they do not introduce significant background noise to the measurement. Furthermore, the low standard deviation of the measurements, along with enhanced ECL intensity (increased sensitivity), of the high-potential measurements in the presence of chloride ions, renders this method suitable for quantitative measurements.

## Conclusion

Bubble formation is usually avoided in electroanalysis and in ECL because it introduces variability and decreases the reliability of electrochemical measurements. However, chlorine bubbles, formed at an electrode in a solution containing chloride ions, extend the spatial and temporal range of ECL events. ECL is a highly localized phenomenon normally restricted to a few micrometers from the electrode surface.

In this work, we have shown that bubble formation enhanced ECL emission of luminol and its derivatives trough two parallel effects: (i) the corona effect of the bubble, which consists of the buildup of reactive oxygen species such as hydroxyl radicals at the gas/liquid interface; (ii) the release of chlorine into the solution, which readily oxidizes luminol in the presence of H_2_O_2_. While the corona effect also triggered ECL in the absence of chloride ions, the second effect only occurred following the oxidation of chloride into chlorine gas bubbles at high anodic potentials (i.e., above 2 V). The subsequent release and dissolution of chlorine in the solution (producing hypochlorite) generated an intense ECL emission at the gas/liquid interface. In addition, bubbles that grow large enough to detach from the electrode surface, continue emitting light during their movement as long as they contain chlorine. Remarkably, we have observed ECL emission from these bubbles up to a remarkable distance of 5 mm away from the electrode surface.

Moreover, the intensity of this bubble-enhanced ECL recorded at 2.4 V was 127.6 times higher than that of the ECL emission recorded at the commonly used potential of 0.7 V. In terms of ECL duration, the emissions recorded at 0.7 V vanished immediately after stopping the electrode polarization. In the case of the electrogenerated bubbles, ECL emissions that lasted for up to 145 s have been observed after electrode polarization. This long-lasting ECL emission is an unusual behavior in the ECL field.

Last, we have shown the utility of this approach for the detection of glucose. Again, the enzymatic detection of glucose performed at 2.4 V in the presence of chloride was 5.2 times higher than that at the same electrode polarized at 0.7 V. This is highly significant in the field of interfacial chemistry and electroanalysis, not only because it provides enhanced signals, but mainly because it represents the first example of ECL produced at the gas/liquid interface, which opens the technique to the study of the dissolution of gases, diffusion of chemical species, and reactions at phase interfaces and in solution.

## Supplementary Material



## Notes

Additional experimental details, L-012 emission in the absence of H_2_O_2_, effect of chloride adsorption on Pt electrodes, O_2_ vs Cl_2_ bubbles, control experiments in the absence of H_2_O_2_, regions of interest for image analysis, ECL stability after electrode polarization, effect of chloride concentration, and glucose control experiments (PDF)
