# Supplementary material for: Enhanced Electrochemiluminescence at the Gas/Liquid Interface of Bubbles Propelled into Solution
Source: J Am Chem Soc. 2024 Aug 2;146(32):22724–35. doi: 10.1021/jacs.4c07566 (PMC12884462; doi:10.1021/jacs.4c07566)
Supplement: Supplementary file 1 [file ja4c07566_si_001.pdf]

Supporting Information

Enhanced electrochemiluminescence at the gas/liquid interface of bubbles propelled into solution

Sara Knežević,[‖,a] Joseba Totoricaguena-Gorriño,[†,a] Rajendra Kumar Reddy Gajjala,[†] Bruno Hermenegildo,[†] Leire Ruiz-Rubio,[†,‡] José Luis Vilas-Vilela,[†,‡] Senentxu Lanceros-Méndez,[†,§] Neso Sojic[‖,*] Francisco Javier del Campo,[†,§,*]

[‖] University of Bordeaux, Bordeaux INP, ISM, UMR CNRS 5255; Pessac, 33607, France.

[†] BCMaterials, Basque Center for Materials, Applications and Nanostructures, UPV/EHU Science Park, 48940 Leioa, Vizcaya, Spain.

[‡] Grupo de Química Macromolecular, Universidad del País Vasco, UPV-EHU, Campus de Leioa, 48940 Vizcaya, Spain.

[§] IKERBASQUE, Basque Foundation for Science, 48009 Bilbao, Spain.

[a] Authors contribute in an equal way to the work.

Email: sojic@u-bordeaux.fr

Email: javier.delcampo@bcmaterials.net

Supporting Information description: additional experimental details (pdf).

Table of contents

Figure S1: L-012 emission in the presence and in the absence of H_2O_2 3
Figure S2: Effect of chloride adsorption on Pt electrodes...4
Figure S3: O_2 vs Cl_2 bubbles 5
Figure S4: Control experiments in the absence of H_2O_2 (images) 6
Figure S5: Control experiments in the absence of H_2O_2 (bar chart) 7
Figure S6: Regions of interest for image analysis..8
Figure S7: ECL stability after electrode polarization...9
Figure S8: Effect of chloride concentration 10
Figure S9: Glucose control experiments 11

Figure S1: L-012 emission in the presence and in the absence of H_2O_2

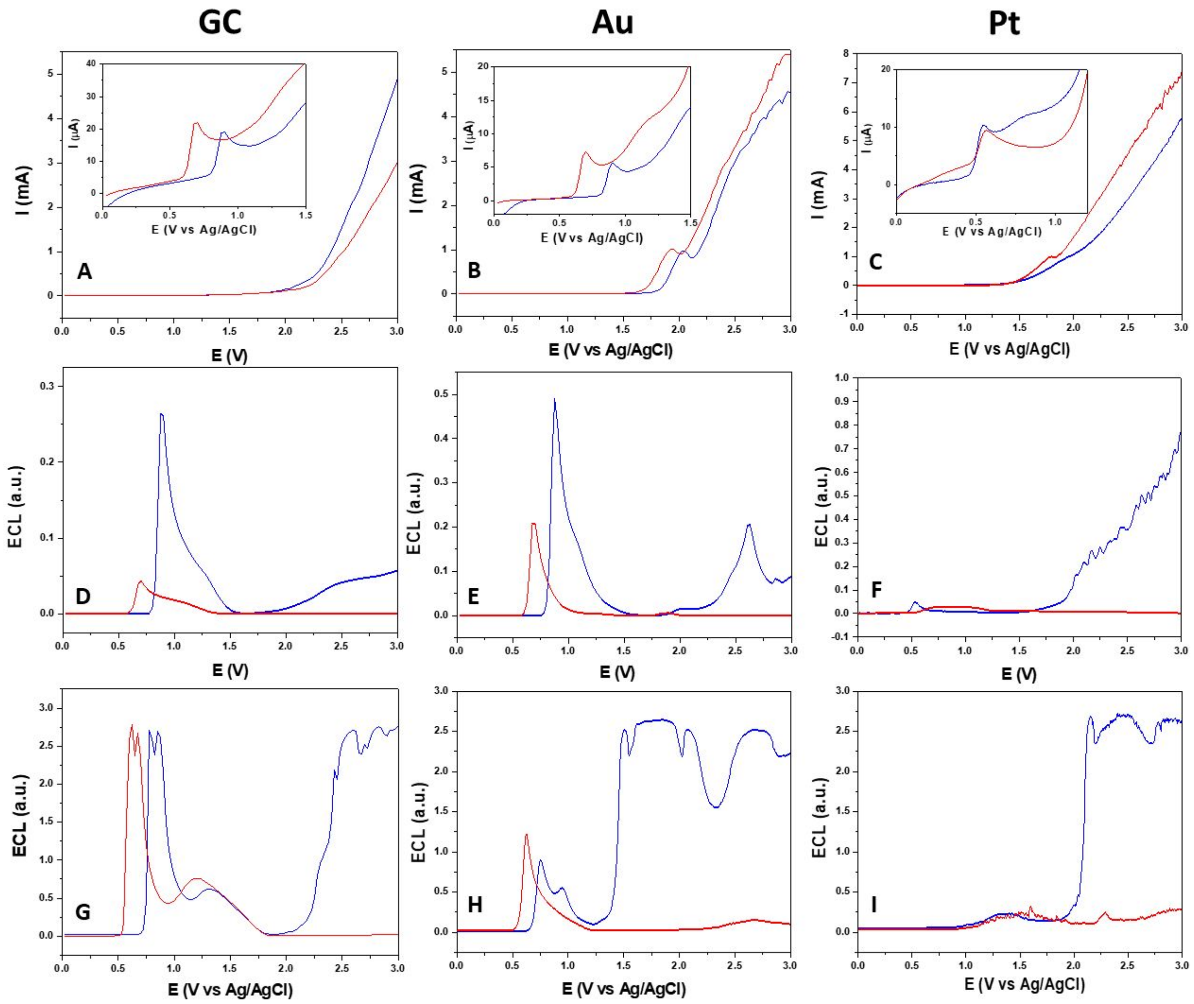

Figure S1. **(A-C)** Linear sweep voltammograms at 0.1 V s^{-1} from 0 V to 3.0 V in 0.2 M PBS (pH = 8) with 50 mM KNO_3 (red) and KCl (blue) on **(A)** GC electrode, **(B)** Au electrode, **(C)** Pt electrode. Insets **(A-C)** show the electrochemical oxidation of L-012 in KNO_3 (red) and KCl (blue) supporting electrolytes on different working electrodes. **(D-I)** Corresponding evolution of the ECL intensity with electrode potential during a linear potential sweep at 0.1 V s^{-1} from 0 V to 3.0 V in 0.2 M PBS (pH = 8), 0.42 mM L-012, 50 mM KNO_3 (in red) or KCl (in blue) and **(D-F)** 0 M H_2O_2 or **(G-I)** 0.5 M H_2O_2 on **(D** and **G)** GC electrode, **(E** and **H)** Au electrode, **(F** and **I)** Pt electrode.

Figure S2: Effect of chloride adsorption on Pt electrodes

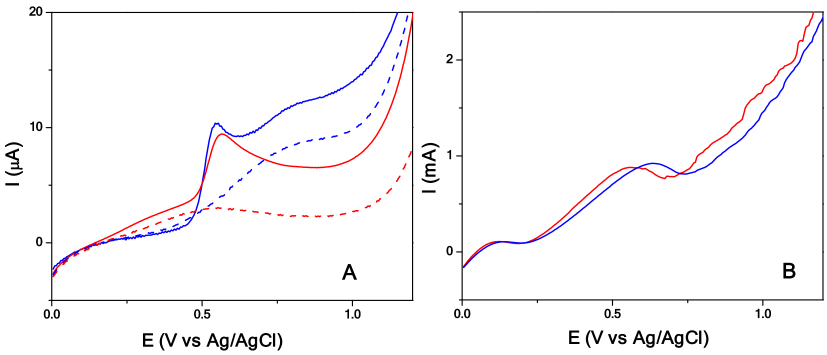

Figure S2. Linear sweep voltammograms at Pt electrode at 0.1 V s^{-1} from 0 V to 1.2 V in 0.2 M PBS pH 8 with 50 mM KNO_3 (red) and KCl (blue) **(A)** containing 0 mM L-012 (dashed lines) and 0.42 mM L-012 (full lines) and **(B)** containing 0.42 mM L-012 and 0.5 M H_2O_2.

Figure S3: O_2 vs Cl_2 bubbles

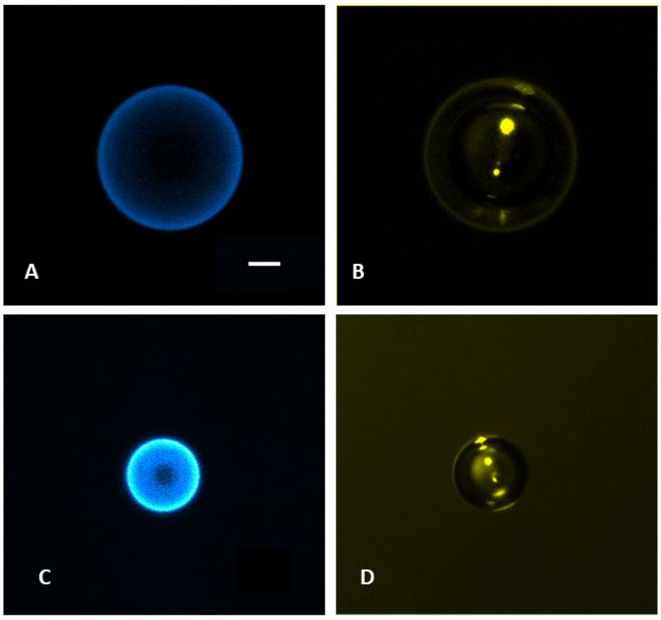

Figure S3. Top view micrographs of the ECL emission **(A** and **C)** and brightfield images **(B** and **D)** of the gas bubbles (O_2 for **(A-B)** and Cl_2 for **(C-D)**) generated at the gold microelectrode in the 0.2 M PBS solution pH 8 containing 0.5 M H_2O_2, 0.42 mM L-012 and 50 mM **(A-B)** KNO_3 and **(C-D)** KCl. Images were taken with the 0.1 s exposure time upon applying a 2.4 V potential pulse. The intensity scales were set to **(A)** 1200-3500 and **(C)** 1200-58000 grey levels. The scalebar is set to 50 µm.

Figure S4: Control experiments in the absence of H_2O_2

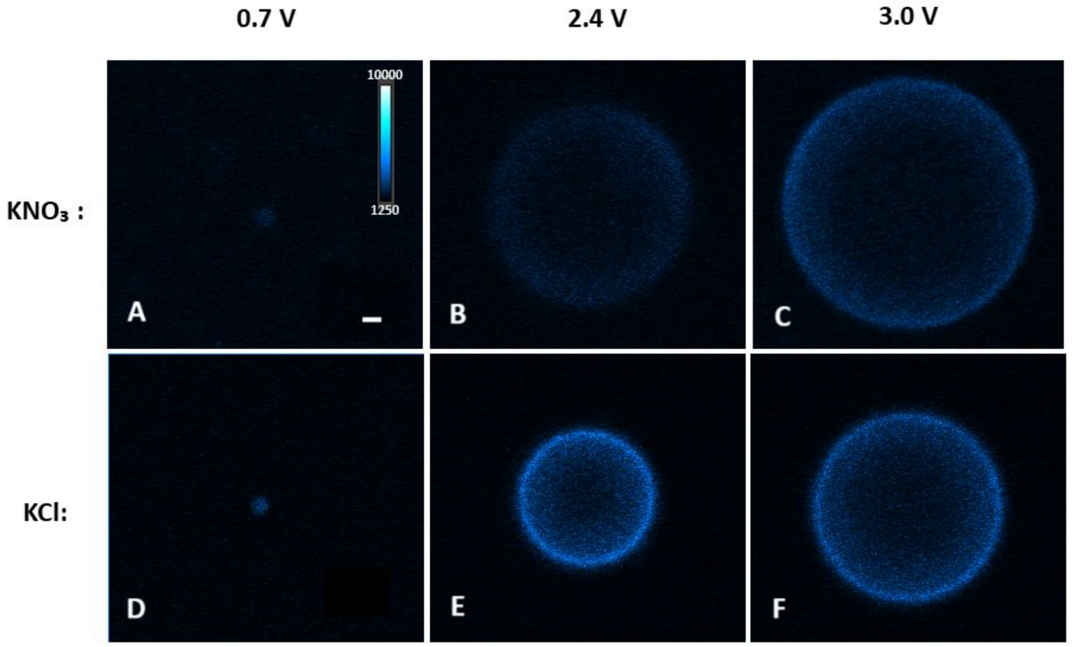

Figure S4. Bottom-view images of the ECL emission at the gold 25-µm disc microelectrode in the 0.2 M PBS solution (pH 8) containing 0.42 mM L-012 and 50 mM **(A-C)** KNO_3 or **(D-F)** KCl. Electrode potential was set to **(A** and **D)** 0.7 V, **(B** and **E)** 2.4 V and **(C** and **F)** 3 V vs Ag/AgCl for 20 s. Images **A** and **D** were recorded with a 3-s exposure time, upon applying the electrochemical potential for 1 s. Images (**B**, **C**, **E** and **F**) were taken with a 1-s exposure time, upon applying the potential for 5 s (when the Cl_2/O_2 bubbles were fully formed). False-color ECL images were coded with the light intensity scales 1200-10000 (shown in **A**). Scale bar: 25 µm.

Figure S5: Control experiments in the absence of H_2O_2

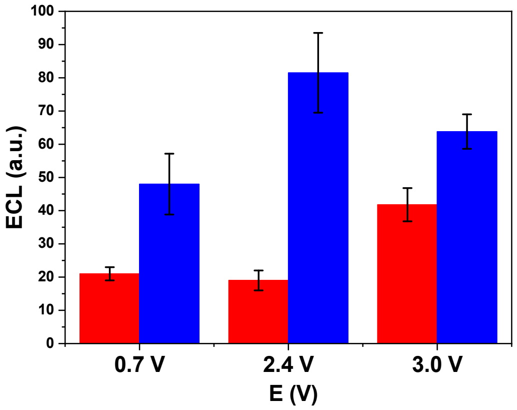

Figure S5. The mean ECL intensity in the presence of 50 mM KNO_3 (in red) or 50 mM KCl (in blue) supporting electrolyte upon applying chronoamperometric pulses of 0.7 V, 2.4 V and 3 V vs Ag/AgCl. The values in the bar chart represent the mean of experiments per conditions as shown in **Figure S4** micrographs performed in triplicate in the presence (in blue) and absence of Cl^- (in red).The error bars indicate ±1 standard deviation from the mean.

Figure S6: Regions of interest for image analysis

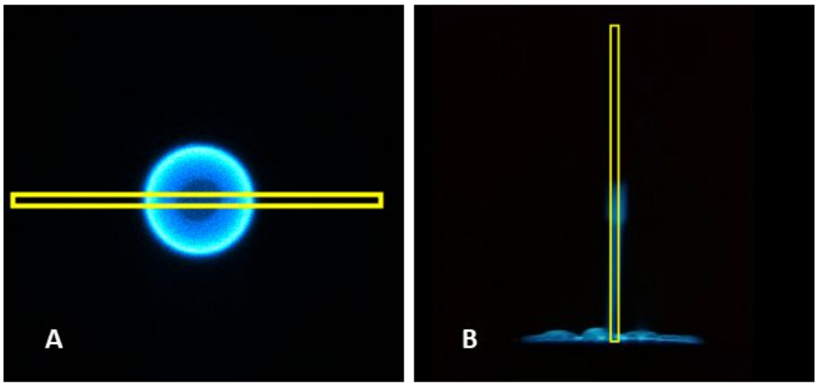

Figure S6. Representative examples of the micrographs with shown rectangular regions of interest, used to extract ECL intensities for the plots in **(A)** Figure 2 (bottom-view) and **(B)** Figure 4 (side-view).

Figure S7: ECL stability after electrode polarization

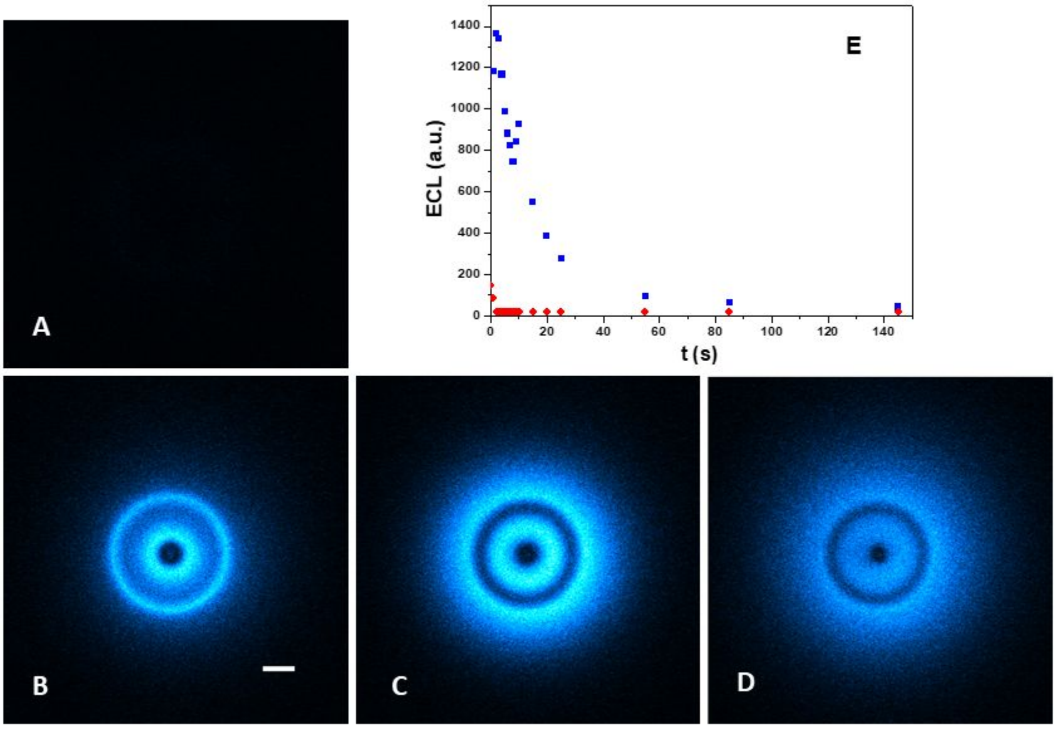

Figure S7. **(A-D)** Top view micrographs of the ECL emission at the gold microelectrode in the 0.2 M PBS solution pH = 8 containing 0.5 M H_2O_2, 0.42 mM L-012 and 50 mM **(A)** KNO_3 and **(B-D)** KCl. Images were captured with a 1 s exposure time, 1 s **(A** and **B)**, 3s **(C)** and 7 s **(D)** after stopping the CA potential pulse of 2.4 V. The intensity scale is set to the range of 1200-15500 units of grey value. The scalebar is set to 50 µm. **(E)** Evolution of the mean ECL intensity with time in the presence of 50 mM KNO_3 (in red) and 50 mM KCl (in blue) supporting electrolyte. Solution composition and electrochemical conditions are consistent with **(A-D)**.

Figure S8: Effect of chloride concentration

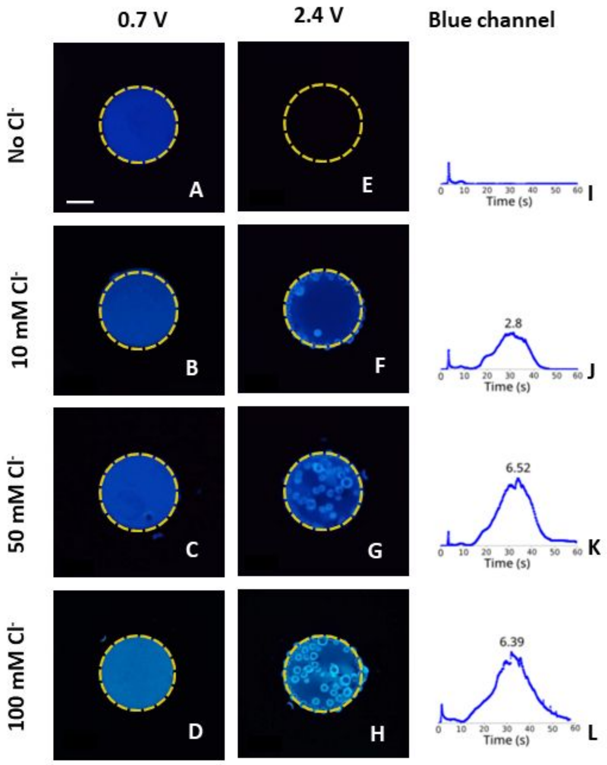

Figure S8. **(A-H)** ECL images and **(I-L)** ECL-t plots extracted from a video recorded at a frame rate of 30 fps during a CV scan at 0.1 V s^{-1} from 0 V to 3.0 V. The images correspond to the 30 ms frames at the potential of **(A-D)** 0.7 V and **(E-H)** 2.4 V. The scalebar is set to 1 mm. The plots **(I-L)** depict the ECL evolution over time during the CV scan. The experiments were conducted in 0.2 M PBS solution pH 8 containing 0.42 mM L-012 and 0.5 M H_2O_2 solutions with varying KCl concentrations: **(A, E and I)** 0 M, **(B, F and J)** 10 mM, **(C, G and K)** 50 mM and **(D, H and L)** 100 mM KCl.

Figure S9: Glucose control experiments

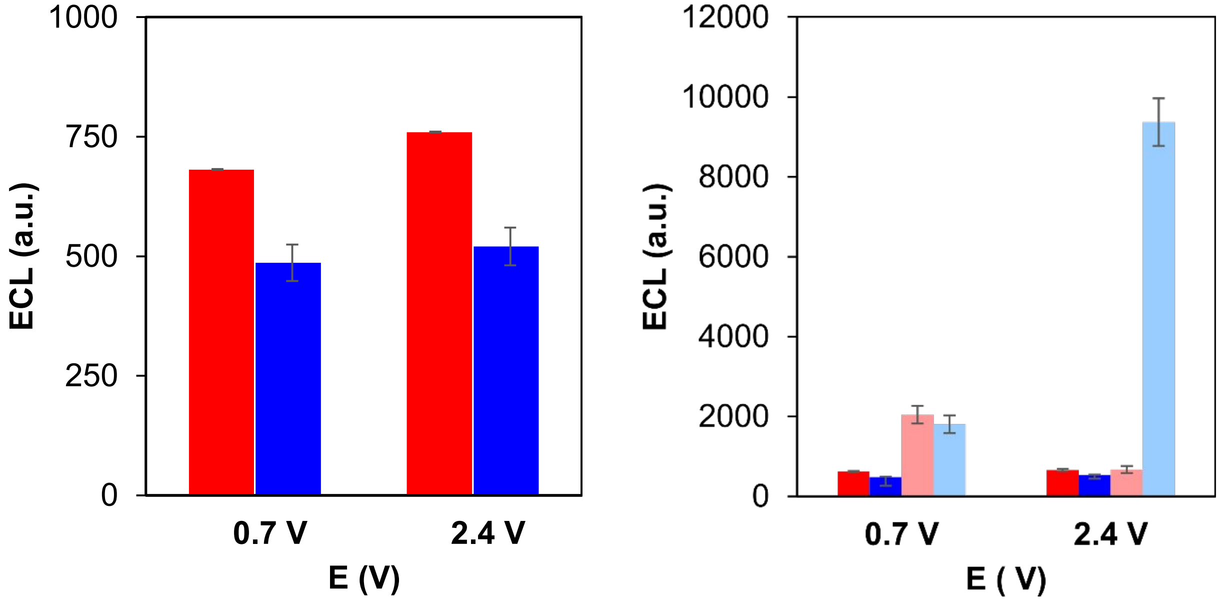

Figure S9. ECL emission at a 3mm GC electrode in 0.2 M PBS solution (pH 8) containing 0.42 mM L-012, 1 mg mL^{-1} glucose oxidase plus 50 mM KNO_3 (red and pink bars) or 50 mM KCl (blue bars). On the left hand side, in the absence of glucose. The right-hand plot shows the data next to the measurements in 1mM glucose.
